# Increasing the Efficiency of a Spintronic THz Emitter Based on WSe_2_/FeCo

**DOI:** 10.3390/ma14216479

**Published:** 2021-10-28

**Authors:** Dinar Khusyainov, Andrey Guskov, Sergei Ovcharenko, Nicolas Tiercelin, Vladimir Preobrazhensky, Arseniy Buryakov, Alexander Sigov, Elena Mishina

**Affiliations:** 1 Department of Nanoelectronics, MIREA—Russian Technological University, 119454 Moscow, Russia; andrey-99@mail.ru (A.G.); ovcharenko@mirea.ru (S.O.); buryakov@mirea.ru (A.B.); sigov@mirea.ru (A.S.); mishina@mirea.ru (E.M.); 2 University of Lille, CNRS, Centrale Lille, Université Polytechnique Hauts-de-France, UMR 8520-IEMN, 59000 Lille, France; nicolas.tiercelin@iemn.fr; 3 Prokhorov General Physics Institute of RAS, 119991 Moscow, Russia; preobr@newmail.ru

**Keywords:** magnetic properties and materials, magneto-optics, optical spectroscopy, spintronics, structural properties, surfaces, interfaces and thin films, terahertz optics, ultrafast lasers, TMDC

## Abstract

We report an increase in terahertz (THz) radiation efficiency due to FeCo/WSe_2_ structures in the reflection geometry. This can be attributed to an absorption increase in the alloy FeCo layer at the input FeCo/WSe_2_ interface due to constructive interference, as well as to the backward transport of hot carriers from FeCo to WSe_2_. In contrast to the transmission geometry, the THz generation efficiency in the reflection is much less dependent on the magnetic layer thickness. Our results suggest a cheap and efficient way to improve the characteristics of THz spintronic emitters with the conservation of a full set of their important properties.

## 1. Introduction

Among the many different types of terahertz (THz) emitters using various physical effects, spintronic ones demonstrate many noticeable advantages, primarily ultra-broad bandwidth but also a high efficiency and ease of control of radiation parameters: polarization [1,2,3,4], amplitude [5,6], phase [7]. Benefits such as an ease of integration and low cost allow spintronic emitters to be integrated into a variety of end-user devices for spectroscopy, medicine and safety. The efficiency increase of THz emitters is always on the agenda as it means a reduction of energy consumption. To increase the THz generation efficiency, different approaches are used.

Most spintronic emitters are a bilayer structure of ferromagnetic/nonmagnetic (FM/NM) nanofilms, where FM = Co, Fe, Ni, and their alloys are the most popular, and where NM = Pt, W, Ir, Pd,… [8]. The inverse spin-Hall effect (ISHE), that is, the conversion of spin current to charge current at the FM/NM interface, is responsible for THz emission in such structures. The terahertz signal amplitude strongly depends on the NM material but changes little when the FM material changes. Hence, the NM materials’ variation is used to improve the emitters’ characteristics [9].

Geometric parameters and the structure design also affect the THz emitter efficiency. The dependence on the FM layer thickness is nonmonotonic and has a pronounced maximum due to the photonic effect in the layers: thin film acts as a Fabry–Pérot cavity and resonantly enhances both the optical pump and terahertz waves. The ratio of constructive interference gain to optical losses determines the position of the efficiency maximum, which is usually around 5 nm. Note that different photonic effects are widely used to enhance THz emitter efficiency [10,11,12]. However, for spintronic emitters, the effects are less efficient due to their very low thickness when compared with the wavelength.

One of the main characteristics affecting the emitter efficiency is the mean free pass λ (MFP) of electrons in the FM, which depends on the material. It was shown in [13] that annealing was a way to enlarge MFP by suppressing disorder scattering: MFP for majority carriers λ_↑_ was estimated to increase by a couple of nanometers for amorphous Co_20_Fe_60_B_20_. An additional option is to use, instead of a two-layered structure, an asymmetric three-layered one in which the outer layers of different NM materials possess opposite signs of ISHE. Along with the opposite signs of the spin current in these layers, this results in almost doubling the THz amplitude [9]. This result indicates that the gain from the total conversion of forward and backward spin currents into terahertz radiation compensates for the losses due to the greater metal thickness of a three-layer structure when compared to a two-layer one.

Recently, an order of magnitude enhancement of THz emission was observed from the Co/MoS_2_ interface [14] due to a giant spin injection of far out-of-equilibrium electrons into atomically thin MoS_2_; the effect was observed in transmission through the Co/MoS_2_ structure.

In this paper, we report an observation of the enhancement of the THz emission efficiency from a FeCo/WSe_2_ structure in reflection geometry. We studied the effect of different thicknesses of alloy FeCo films and WSe_2_ flakes. We analyze the mechanism of THz radiation emission and show that enhancement can be even stronger in reflection than in transmission geometry.

## 2. Materials and Methods

The alloy FeCo films with 40 nm and 20 nm thicknesses (in the following FeCo40 and FeCo20, respectively) were deposited on a glass substrate by RF sputtering technology. The magnetic properties of the samples were tested for in-plane orientations of the magnetic field by the magneto-optical Kerr effect (MOKE). The hysteresis loops measured along two perpendicular directions are rectangular and almost equal for both samples, revealing their in-plane magnetic isotropy.

**Figure 1:**
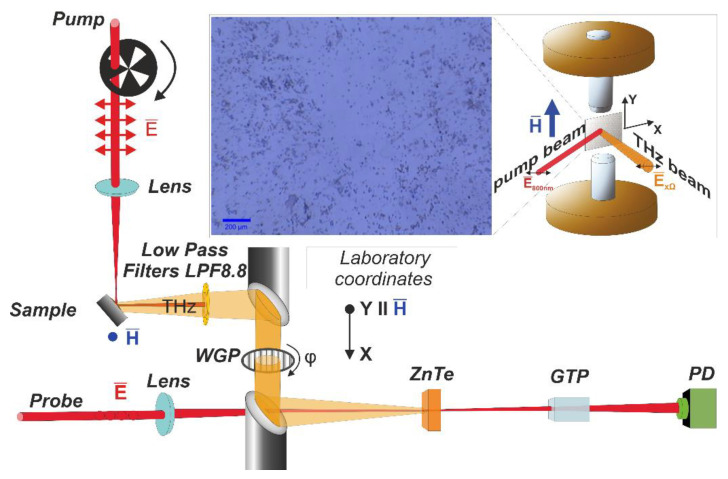
Schematic representation of the set−up, where WGP is the wire-grid polarizer, GTP is the Glan Taylor polarizer, and PD is the photodiode. Insets: optical micrograph of the sample and sample geometry in the magnetic field.

Transition metal dichalcogenide (TMD) WSe_2_ flakes were deposited on top of the magnetic layer using standard mechanical exfoliation techniques from the commercial bulk crystal. The flakes were deposited on the half of the FeCo films to directly compare in the same experiment the efficiency of the WSe_2_-covered and bare FeCo film THz emitter. The optical image of the surface of the fabricated WSe_2_/FeCo structures is shown in Figure 1, inset. The area covered by the flakes, estimated from the images, is about 10% for all samples. The flake thickness was measured by an atomic force microscope. The average thickness was estimated as 45 nm for FeCo20/WSe_2_ and 37 nm for FeCo40/WSe_2_.

The samples were mounted on a nonmagnetic holder between two electromagnet cores oriented vertically, which allowed one to apply fields of up to 5 kOe in the plane of the sample along the Y axis of the laboratory frame (Figure 1, inset).

For the comparison of the THz generation efficiency, the reference sample was used: the mother bulk WSe_2_ crystal with the parameters described in Ref [15]. All measurements were performed in the reflection geometry at room temperature. The choice of the reflection geometry was based on preliminary calculations using the model presented in [16] and showed that, in the transmission geometry, THz radiation was strongly absorbed by 20 nm and 40 nm FeCo films.

The THz emission characteristics were studied using a time-domain spectroscopy scheme (Figure 1). For pumping, an amplified Titanium:Sapphire-based femtosecond laser system (TiF-20F, Avesta project, Moscow, Russia) was used and provided a 35 fs laser pulse with a repetition rate of 3 kHz at a central wavelength of 800 nm. The output optical radiation was split into two beams: an optical pump pulse and an optical probe pulse. An optical pump pulse was focused onto the sample surface at 45 degrees’ incidence, with the energy density ~1 mJ/cm^2^. The polarization of both the pump and probe beams was linear. The polarization of the pump and probe beams was parallel to the *X* and *Y* axes of the laboratory frame, respectively (Figure 1, inset). The THz radiation generated in the sample in reflection geometry was collimated by a parabolic mirror, and then, via the second parabolic mirror, it was focused onto the surface of the <110> ZnTe crystal detector. The optical pump radiation was cut off by a filter. The probing beam, passing through the delay line, was focused onto the surface of the ZnTe crystal detector as well and was spatially overlapped with the THz beam. After interacting with the detector crystal, the probe beam passed through a crossed polarizer (zero-point electro optical regime measurements [17]). In accordance with Ref. [18], the polarization of the probe beam was set parallel to the [1] axis of the ZnTe detector crystal, which allowed one to detect the X component of the THz field (Ex_Ω_). 

In our experiment, the delay time was changed for fixed experimental parameters: the magnetic field, pump pulse energy density and wire-grid polarizer (WGP) rotation angle φ. The data obtained were processed in different ways in relation to the goal of the experiment. First, using Fast Fourier Transform (FFT), the spectra of the emitted radiation were obtained. Then, the peak-to-peak values of the THz signal, proportional to the instantaneous value of the electric field in the THz pulse, were measured using the software processing of the time domain dependences. Rotating the WGP allows one to determine the polarization of THz radiation (for details, see [2]).

## 3. Results

**Figure 2:**
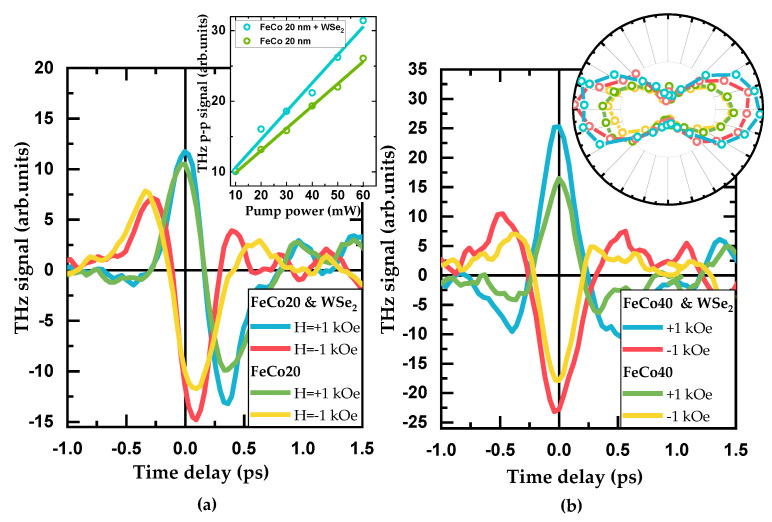
Terahertz emission from bare and WSe_2_−covered FeCo films at opposite directions of the magnetic field: (**a**) FeCo20, (**b**) FeCo40. Insets: (**a**) power dependence and (**b**) polarization dependence of the raw peak−to−peak signal.

The time domain dependences of the THz signal at different values of the magnetic field for the WSe_2_-covered and bare FeCo films are shown in Figure 2. For FeCo40, the dependences are “classical” for spintronic emitters: a change in the sign of the magnetizing field inverts the THz pulse for both the WSe_2_-covered and bare FeCo40 films. The TMD cover increases the THz signal by up to 30%. For FeCo20, the magnetic field inversion results in a more complicated change of the pulse shape accompanied by its phase shift. The TMD cover increases the THz signal by up to 20%.

The insets in Figure 2 show the details of the THz emission: the power dependences and polarization dependences of the peak-to-peak THz signal. These dependences are very similar for all samples, so we present examples for only one sample. It is clearly seen that the main characteristics of the THz emission are completely determined by the ferromagnetic layer. The TMD cover increases the signal by 20% in average, keeping the polarization and power dependences unchanged.

Linear dependences on the excitation power (Figure 2a, inset) are typical for spintronic emitters, and this means no demagnetization of the sample under the influence of the maximum pump power [9].

Polarization dependences of the THz signal (Figure 2b, inset) are also typical for spintronic emitters and indicate the linear polarization of the THz wave. It is determined by a magnetic field direction perpendicular to it and rotates with the magnetic field direction, indicating its spin-based origin. The same behavior has been observed in Ref. [14], but in transmission geometry.

**Figure 3:**
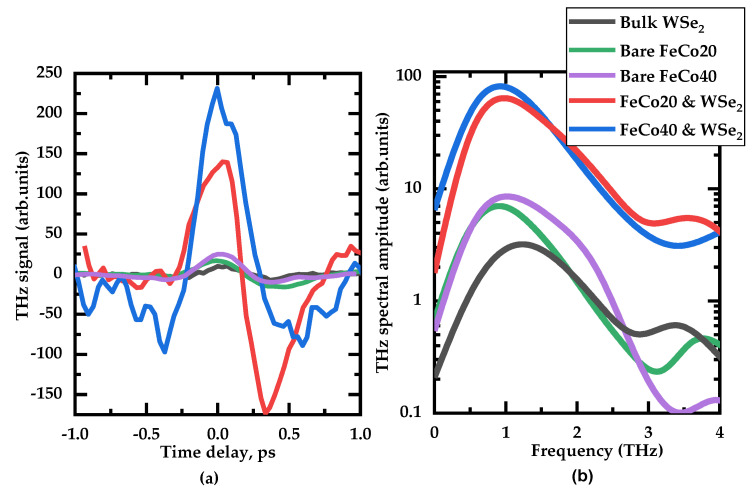
(**a**) THz time−domain signal generated by various structures and (**b**) correspondent spectra.

To compare the spectral characteristics and shed light on the source of the THz emission, we compare the THz signals of all samples (taking into account the coverage factors for WSe_2_-covered FM films), and the results are shown in Figure 3. Bulk WSe_2_ reveals the lowest signal, which gives reasons to neglect the separate contribution of the flakes covering the spintronic emitters as a source of the enhanced THz signal. Among the two samples of bare FeCo, FeCo40 exhibits a two-fold higher THz signal than FeCo20. The experimental 10% TMD filling factor leads to around a 20 percent enhancement of the THz signal in comparison with bare ferromagnetic films. This gives an estimate for efficiency enhancement as an order of magnitude, for a 100% TMD filling factor.

The spectra of all the samples studied are equal within the noise level, which emphasizes the fact that the TMD layer only enhances the signal, not changing its spintronic nature.

## 4. Discussion

In the following, we would like to clarify two questions: (i) whether the THz radiation mechanism is the same in both transmission and reflection geometry; (ii) whether the reflection geometry can provide a higher gain of THz radiation emission and why.

The electronic density of states in a ferromagnet is split into majority and minority bands due to the exchange interaction. After the photon energy is absorbed, a certain amount of majority and minority electrons are excited to different energies above the Fermi level with unequal velocities 〈v〉 and lifetimes τ. Majority electrons have larger velocities and longer lifetimes than minority ones. An important parameter for spin transport is a mean free pass (MFP) λ = 〈v〉∙τ. Based on Refs. [18,19] for the FeCo alloy, we can take for the estimation of the majority electrons MFP λ_↑_~10 nm.

In [19,20], for the FM/NM structure, a new type of spin transport was suggested when the film thickness was comparable to the MFP for the majority carriers. The effect was considered in transmission geometry. Classically, for thick films ($d\gg \;\lambda_{↑}$), ordinary diffusive transport takes place, and demagnetization occurs due to spin-flip transitions of majority to minority electrons within the diffusion through a temperature gradient. For very thin films (d<λ_↑_), ballistic transport takes place. For an intermediate thickness of the FM layer, when d~ λ_↑_, superdiffusive transport takes place, for which demagnetization occurs due to the penetration of majority electrons into the NM metal, while minority electrons are stuck in the FM. In the transmission, these effects result in a strong dependence of the THz generation efficiency on the FM thickness: it decreases by an order of magnitude when the thickness of the FM layer increases from 2 to 8 nm [16].

**Figure 4:**
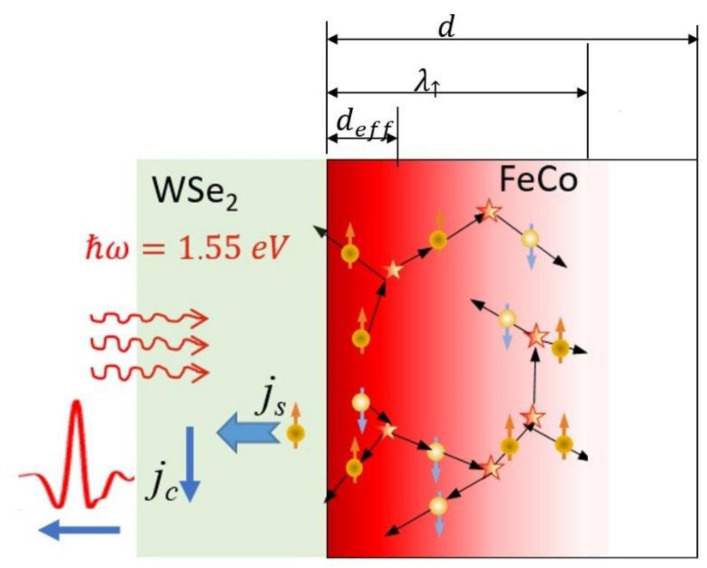
Schematic cross-section views of the structure WSe_2_/FeCo and representation of the backflow spin current arising in FM due to inelastic scattering. d—the FM layer thickness, $\lambda_{↑}$—the majority electrons MFP, $d_{eff}$—the maximal distance, which is necessary for scattered electrons to overcome and reach the WSe_2_/FeCo boundary in the ballistic or/and superdiffusion regime.

In order to observe THz emission in reflection, we need a backflow spin current, which transfers into a charge current via ISHE. Obviously, a backflow spin current arises in FM due to inelastic scattering (See Figure 4). According to [19], an excited electron undergoing an inelastic scattering with another electron will transfer part of its energy to the other one, generating a cascade of electrons. In Ref. [19], only transmission geometry is considered, but the effect has to exist in the backward direction as well. An experimental confirmation of the effect can be found in [9] for a NM/FM/NM THz emitter. A numerical confirmation of the effect can be found in [20]. Calculations of the magnetization change map under conditions of superdiffusive transport in the Pt/Ni/Pt/Al reveal a strong demagnetization of the Ni layer in the vicinity of interfaces and a magnetization of nonmagnetic Pt layers. Surprisingly, the strongest demagnetization/magnetization is observed not at the forward interface but at the backward (or input) interface. This issue, which has not been discussed in [20], is important for the consideration of the reflection geometry.

Thus, we answer question (i): yes, the mechanism for THz radiation generation is the same in both transmission and reflection geometry.

The number of excited electrons in the FM film depends on the input laser fluence and absorption coefficient. The latter is constant across the film, but the former decreases exponentially according to the Bouguer–Lambert–Beer law. In the input subsurface slabs, the absorbed light intensity and correspondent hot electron density determined by a cross-section of the appropriate process are the highest. On the other hand, for backward-scattered electrons, the distance $d_{eff}$ to the input surface is the smallest, and electrons (preferably with majority spins) can reach the surface in both a ballistic and superdiffusive regime, since $d_{eff}\leq \lambda_{↑}$. Obviously, near the FeCo/TMD input interface, we lose in the number of electrons per incident photon scattered in FeCo in the reflection direction [21,22], but we gain to a large extent in the absorbed optical energy (and, therefore, in the number of generated hot electrons and their energy). In total, we get a gain in the density of the spin-polarized current.

If there is no cap NM layer, where ISHE takes place effectively, the prize is not so big, but it exists. Thus, we have to answer question (ii) positively.

Finally, let us consider the WSe_2_ layer on top of the FM film. It was shown in [23] that for the MoS_2_/FM structure, the superdiffusive transport of hot non-equilibrium electrons in transmission geometry provided the spin-current generation mechanism. Apart from ISHE, which provides an efficient THz pulse generation, the MoS_2_ plays an additional role. The semiconductor’s bandgap only works as a filter for the high-energy carriers where the population is almost fully spin-polarized; this leads to an extremely high spin polarization of the current injected into the semiconductor.

**Figure 5:**
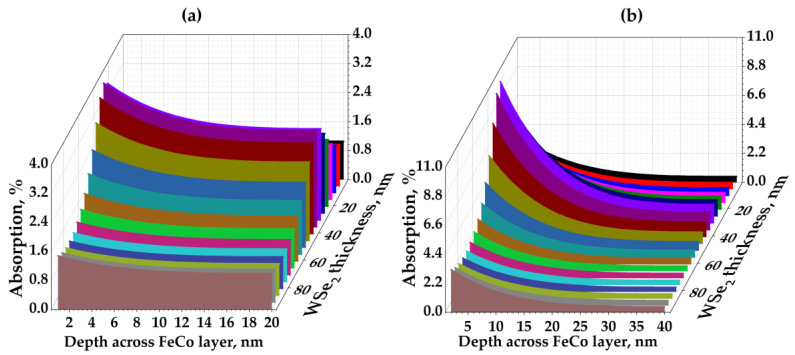
Calculated dependences of the optical absorption on the depth across the FM layer and on the thickness of the TMD layer in (**a**) FeCo20/WSe_2_ and (**b**) FeCo40/WSe_2_.

In [23], a monolayer is considered. In our case with a thicker WSe_2_ layer, the effect not only persists but can also be enhanced due to the formation of a more efficient Fabry–Pérot cavity in the WSe_2_/FM structure. To prove this, we performed model calculations (using COMSOL Multiphysics) of the field absorbed in the subsurface layers of the FM film for different thicknesses of the WSe_2_ layer. Figure 5a shows the calculated dependences of the laser radiation absorption on the depth across the FM layer (the distance from the sample surface) and on the thickness of the TMD layer in the FeCo20/WSe_2_ (Figure 5a) and FeCo40/WSe_2_ (Figure 5b) samples. 

For FeCo20/WSe_2_, the maximum absorption of about 4% for the laser radiation propagated through a 2-nm FeCo depth and was observed for a WSe_2_ thickness of 36 nm. This thickness is very close to the experimental value of 45 nm for the average WSe_2_ thickness. The calculated absorption of the incident radiation for a 45-nm thick WSe_2_ is about 3%. For FeCo40/WSe_2_, the maximum absorption is much higher and is about 11% for a 2–3 nm-FeCo depth, and it is observed for a WSe_2_ thickness of 31 nm. The calculated absorption of the incident radiation for an average experimental WSe_2_ thickness of 37 nm is about 10%. Thus, absorption in the FM subsurface depends on the thickness of the whole FM layer and is twice higher in a thicker film.

Generally, the optical interference in a Fabry–Perot cavity formed by FeCo/WSe_2_ structures provides a significant increase in the optical absorption and electric field distribution inside the FM layer. The latter, in turn, provides a higher density of hot electrons [24,25], as well as a higher spin current and THz signal [16]. 

Thus, in the reflection, the thicker FM layer provides a higher THz generation efficiency than the thinner FM film, in contrast to the transmission geometry. This is fully confirmed by our experimental results.

## 5. Conclusions

In conclusion, we have studied the simplest spintronic emitter, which is the nanosized FeCo film. We used a reflection geometry, which makes the emitter characteristics much less dependent on its exact thickness. We covered the film with WSe_2_ flakes in the simplest way by exfoliating them on top of the magnetic film from a bulk crystal. An increase in the efficiency of a spintronic THz emitter is shown due to the coating with WSe_2_ flakes. This makes the proposed technique a method for the cheap production of efficient THz emitters, which possess an important property of spintronic emitters, such as a simple polarization control. An analysis of the possible mechanisms of the observed enhancement brought us to the conclusion that this is a backward superdiffusive and/or ballistic spin-polarized current generation upon a femtosecond optical pulse excitation. This effect has never been observed in reflection geometry, in which it can be even more efficient than in transmission geometry due to a higher optical fluence and a lower effective thickness at the input interface.

## Data Availability

The data presented in this study are available on request from the corresponding author.
